# HOCOMOCO in 2024: a rebuild of the curated collection of binding models for human and mouse transcription factors

**DOI:** 10.1093/nar/gkad1077

**Published:** 2023-11-16

**Authors:** Ilya E Vorontsov, Irina A Eliseeva, Arsenii Zinkevich, Mikhail Nikonov, Sergey Abramov, Alexandr Boytsov, Vasily Kamenets, Alexandra Kasianova, Semyon Kolmykov, Ivan S Yevshin, Alexander Favorov, Yulia A Medvedeva, Arttu Jolma, Fedor Kolpakov, Vsevolod J Makeev, Ivan V Kulakovskiy

**Affiliations:** Vavilov Institute of General Genetics, Russian Academy of Sciences, 119991 Moscow, Russia; Institute of Protein Research, Russian Academy of Sciences, 142290 Pushchino, Russia; Vavilov Institute of General Genetics, Russian Academy of Sciences, 119991 Moscow, Russia; Faculty of Bioengineering and Bioinformatics, Lomonosov Moscow State University, 119991 Moscow, Russia; Faculty of Bioengineering and Bioinformatics, Lomonosov Moscow State University, 119991 Moscow, Russia; Vavilov Institute of General Genetics, Russian Academy of Sciences, 119991 Moscow, Russia; Altius Institute for Biomedical Sciences, 98121 Seattle, WA, USA; Vavilov Institute of General Genetics, Russian Academy of Sciences, 119991 Moscow, Russia; Altius Institute for Biomedical Sciences, 98121 Seattle, WA, USA; Vavilov Institute of General Genetics, Russian Academy of Sciences, 119991 Moscow, Russia; Moscow Institute of Physics and Technology, 141700 Dolgoprudny, Russia; Institute of Biochemistry and Genetics of the Ufa Federal Research Centre of the Russian Academy of Sciences, 450054 Ufa, Russia; Skolkovo Institute of Science and Technology, 121205 Moscow, Russia; Institute for Information Transmission Problems of the Russian Academy of Sciences, 127051 Moscow, Russia; Department of Computational Biology, Sirius University of Science and Technology, 354340 Sirius, Krasnodar region, Russia; Biosoft.Ru LLC, 630090 Novosibirsk, Russia; Vavilov Institute of General Genetics, Russian Academy of Sciences, 119991 Moscow, Russia; Johns Hopkins University School of Medicine, Baltimore, MD 21205, USA; Research Center of Biotechnology RAS, Russian Academy of Sciences, 119071 Moscow, Russia; Donnelly Centre, University of Toronto, Toronto, Ontario M5S 3E1, Canada; Department of Computational Biology, Sirius University of Science and Technology, 354340 Sirius, Krasnodar region, Russia; Bioinformatics Laboratory, Federal Research Center for Information and Computational Technologies, 630090 Novosibirsk, Russia; Vavilov Institute of General Genetics, Russian Academy of Sciences, 119991 Moscow, Russia; Moscow Institute of Physics and Technology, 141700 Dolgoprudny, Russia; Institute of Biochemistry and Genetics of the Ufa Federal Research Centre of the Russian Academy of Sciences, 450054 Ufa, Russia; Vavilov Institute of General Genetics, Russian Academy of Sciences, 119991 Moscow, Russia; Institute of Protein Research, Russian Academy of Sciences, 142290 Pushchino, Russia; Laboratory of Regulatory Genomics, Institute of Fundamental Medicine and Biology, Kazan Federal University, 420008 Kazan, Russia

## Abstract

We present a major update of the HOCOMOCO collection that provides DNA binding specificity patterns of 949 human transcription factors and 720 mouse orthologs. To make this release, we performed motif discovery in peak sets that originated from 14 183 ChIP-Seq experiments and reads from 2554 HT-SELEX experiments yielding more than 400 thousand candidate motifs. The candidate motifs were annotated according to their similarity to known motifs and the hierarchy of DNA-binding domains of the respective transcription factors. Next, the motifs underwent human expert curation to stratify distinct motif subtypes and remove non-informative patterns and common artifacts. Finally, the curated subset of 100 thousand motifs was supplied to the automated benchmarking to select the best-performing motifs for each transcription factor. The resulting HOCOMOCO v12 core collection contains 1443 verified position weight matrices, including distinct subtypes of DNA binding motifs for particular transcription factors. In addition to the core collection, HOCOMOCO v12 provides motif sets optimized for the recognition of binding sites *in vivo* and *in vitro*, and for annotation of regulatory sequence variants. HOCOMOCO is available at https://hocomoco12.autosome.org and https://hocomoco.autosome.org.

## Graphical Abstract

**Graphical Abstract:**
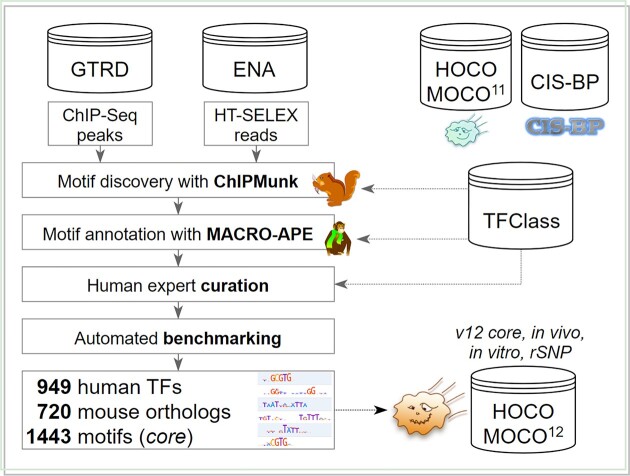

## Introduction

Computational annotation of transcription factor binding sites (TFBS) remains an essential pillar supporting the dome of gene regulation studies. The most common context is the recognition of individual TFBS in genome regulatory regions (1), e.g. as supportive evidence for transcription factor (TF) target genes (2). Predicted TFBS can reveal the genomic locations and the structure of regulatory regions and thus provide information on the composition of transcriptional regulatory complexes (3,4). In synthetic biology, the information on possible locations of TFBS is needed to create biologically neutral spacers in designed CRISPR-Cas guide RNAs for controlled transcription modulation of target genes (5). Finally, TF binding motifs are widely used to interpret regulatory sequence variants within TFBS (6), including non-coding single-nucleotide polymorphisms associated with predisposition to hereditary syndromes (7–10) and somatic mutations occurring in stem cells and cancer (11,12).

Lots of advanced approaches to model and predict TFBS using high-throughput data were presented in the past decade (13–18) yet the classic position weight matrices (PWMs) (19), also called the position-specific scoring matrices, remain the off-the-shelf solution that is widely applied in practice. More than ten years ago we introduced the HOCOMOCO collection of transcription factor binding models. Since then, HOCOMOCO became one of the key resources in the field along with CIS-BP (20) and JASPAR (21), and powered multiple studies in human and mouse gene regulation and epigenetics (22–25). However, the last release of HOCOMOCO was dated 2018, and a recent accumulation of high-throughput data and improvements in transcription factor annotation demanded substantial upgrading of the collection. It became both necessary and possible to cover more transcription factors and alternative binding motif subtypes. The wealth of data also made it possible to improve the motif quality through human expert curation based upon the significantly expanded compendium of studied TFs and the volume of available experimental information for each of the TFs. Here we present HOCOMOCO v12, which was rebuilt from scratch utilizing *in vivo* and *in vitro* high-throughput data on TF binding obtained with ChIP-Seq and HT-SELEX, respectively. By systematic reanalysis and careful curation, we assembled an updated catalog of binding motifs for 949 human TFs and 720 mouse orthologs. The motifs were computationally benchmarked in different scenarios, including recognition of sites bound *in vivo*, *in vitro*, and detection of altered TF binding at regulatory single-nucleotide variants and polymorphisms (rSNPs).

## Materials and methods

### HOCOMOCO transcription factors master list

The first step of the HOCOMOCO reassembly was populating the catalog of human and mouse transcription factors. We have parsed, merged, and verified the gene-ID mapping of the existing TFClass classification (26,27) available online (https://tfclass.bioinf.med.uni-goettingen.de/, https://genexplain.com/tfclass/huTF_classification_Classes.html) for orthologous human and mouse TFs. The classification was extended by adding ten methyl-CpG-binding domain proteins and supplemented with external protein IDs. The resulting HOCOMOCO master list (see Supplementary Table ST1) contains 2681 entries describing 1104 human + mouse orthologous pairs and 473 human-only TFs. Additionally, we annotated the master list with the information from (28) highlighting 1378 (human) and 921 (mouse) proteins with strong documented evidence of being the genuine DNA-binding transcription factors.

### Overview of the HOCOMOCO pipeline

**Figure 1.**
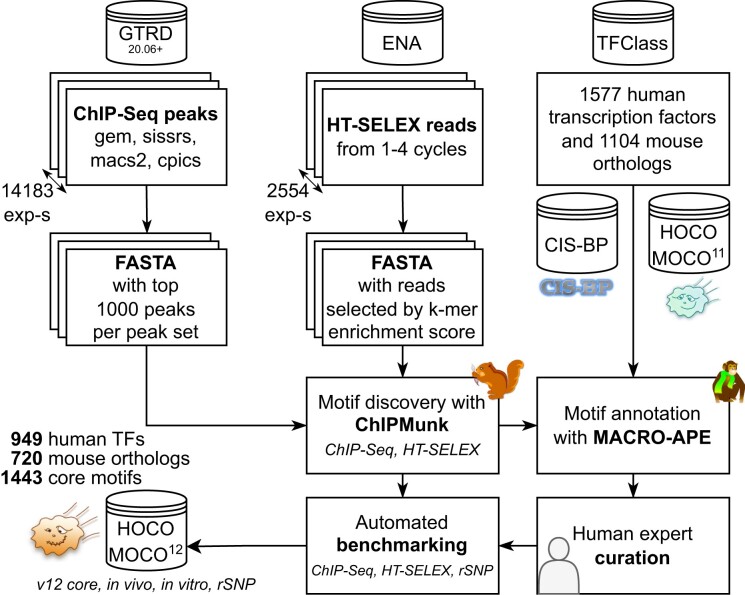
An overview of the HOCOMOCO v12 pipeline. ChIP-Seq peaks from GTRD and HT-SELEX reads from ENA are preprocessed and supplied to motif discovery with ChIPMunk, followed by motif annotation with MACRO-APE using known CIS-BP and HOCOMOCO v11 motifs as reference. The final collection is assembled by human expert curation and automated benchmarking.

An overview of the pipeline used for constructing HOCOMOCO is shown in Figure 1. To construct this release, we utilized two major sources of high-throughput data on DNA-protein recognition *in vitro* and *in vivo*: peak calls from chromatin immunoprecipitation followed by deep sequencing (ChIP-Seq) and high-throughput systematic evolution of ligands by exponential enrichment (HT-SELEX). ChIP-Seq peaks were extracted from the GTRD database (29). HT-SELEX reads obtained in (30–32) were downloaded from the European Nucleotide Archive (ERP001824, ERP001826, PRJEB14744, PRJEB9797). After preprocessing (see the details below), the resulting sequences were supplied to ChIPMunk motif discovery software (33,34), followed by (I) automated annotation of resulting motifs by similarity with known motifs present in CIS-BP (20) and HOCOMOCO v11 (35) using MACRO-APE (36) within the TFClass family and subfamily, (II) human expert curation, and (III) automated benchmarking. The benchmarking results were used to re-visit and improve curation of particular motif subtypes, and then assemble the final motif collection.

### Experimental data overview

**ChIP-Seq data**. ChIP-Seq and ChIP-exo peak sets were extracted from GTRD (mainly from ver. 20.06 and partly from ver. 21.12), see Supplementary Table ST2. GTRD provides peak calls from four different peak calling tools: macs2, gem, pics, sissrs (37–40). In total, the source data from the 14 183 experiments covered 1022 human and 468 mouse TFs with more than 50 thousand peak sets, more than doubling the size of the ChIP-Seq data volume used for assembling HOCOMOCO v11.

**HT-SELEX data**. We used the data from 1810 traditional HT-SELEX experiments: 546 experiments from Jolma *et al.*, 520 from Yang *et al.* and 744 from Yin *et al.* (30–32). In addition, we considered the results of 744 methyl-CpG HT-SELEX experiments of Yin *et al.* The comprehensive list of the datasets is provided in the Supplementary Table ST3.

### Motif discovery

#### Motif discovery from ChIP-seq peaks

**Data preparation**. For each peak set, we prepared subsets of the top 1000 peaks ranked (a) by the peak height and (b) if provided by a particular peak calling tool, peak statistical significance. Peak regions were used ‘as is’ except gem, for which 301 bp regions around peak summits were taken for analysis.

**Motif discovery**. For each peak subset, we generated fasta files using bedtools getfasta and ran ChIPMunk (33) twice searching for short single-box motifs and longer motifs of arbitrary structure. From the ChIPMunk output, we excluded motifs from the consequent analysis if constructed from less than 50 words or covering <25% of the peak set. In total, >300 thousand ChIP-Seq motifs entered the curation stage.

#### Motif discovery from HT-SELEX reads

**Data preparation**. Reads from later cycles of HT-SELEX are likely to resemble the consensus better. However, in some experiments, the later cycles are over-enriched with identical consensus reads, while extra information might be available in the earlier cycles. Thus, two read sets were defined for each HT-SELEX experiment: full (all experiment cycles combined) and late cycles only (starting from the third cycle). In each case, the reads from the selected cycles were pooled and the sequences were ranked by 5-mer enrichment against the dinucleotide-shuffled background. The ‘singleton’ reads that were found only once in a pooled dataset were removed. To allow the motif occurrences to partially overhang the constant HT-SELEX adapters, each sequence was extended by 5′-NNX_1_- and -X_2_NN'-3′, where X_1,2_ are the constant nucleotides of experiment-specific adapters flanking the HT-SELEX random inserts. For each read set, we generated up to four sequence sets using top 1000, 2500, 5000 and 10 000 reads, or all available unique reads if there were not enough available. In total, up to eight sequence sets were produced from a single HT-SELEX experiment.

**Motif discovery**. As HT-SELEX sequences are 10–100 times shorter than those from ChIP-Seq, it was computationally feasible to use the dinucleotide version of ChIPMunk (34) to better account for the background composition, although in the end, we have produced mononucleotide PWMs from the resulting sequence alignments. In total, nearly 20 thousand motifs from HT-SELEX entered the curation stage.

Please refer to the Supplementary Table ST4 for exact ChIPMunk command-line parameters.

### Motif annotation and human expert curation

To facilitate human curation, the motifs were annotated with known motifs of the same TF, TFClass subfamily, and family, using the MACRO-APE similarity comparison tool (36) and HOCOMOCO v11 (35) along with CIS-BP (20) as the reference motif collections. CIS-BP served as a source of diverse putative motifs when checking the motif similarity at the TF family level. In turn, HOCOMOCO v11 was used as a direct primer for curation to pre-sort and group the candidate motifs based on similarity to previously annotated subtypes, if any. As in a previous large-scale benchmarking study (41) the motif performance was not affected by the motif information content, the latter was not used as a curation criterion.

#### Overview of the curation workflow

The motif logos of the annotated motifs of each TF were independently inspected by two junior curators who decided upon grouping motifs into known subtypes, introducing new subtypes, and discarding non-relevant patterns or common artifacts, such as low-complexity poly-A patterns or ETS motifs commonly found in ChIP-Seq peaks of other TF families. The curation decision was guided by motif annotation and TF information from HOCOMOCO v11, CIS-BP and JASPAR, taking into account known motifs of the TFClass structural family and subfamily. Particularly, a motif subtype was introduced when similar alternative motifs were discovered recurrently in multiple ChIP-Seq peak sets, or in both ChIP-Seq and HT-SELEX, or for related TFs of the same family. The curation results from junior curators were assessed and further refined by two senior curators, and the disagreements were resolved on a case-by-case basis. As a general rule, vague DNA patterns (e.g. unstructured low-complexity poly-T tracts) found in a single experiment with missing external confirmation (considering CIS-BP, JASPAR, or publications which yielded the source data) were discarded, while well-defined motifs (e.g. having high information content core regions with less conserved flanking regions) and motifs resembling the known patterns of related TFs were kept. In this setting, Zinc-finger TFs proved to be the most difficult case due to their affinity to genomic repeats (42) making it non-trivial to distinguish genuine binding sites from common artifactual signals such as various parts of ALU repeats. In the end, many of Zinc-finger TFs' motifs received the C quality due to being found only in a single experiment.

#### Curating distinct motif subtypes

HOCOMOCO v11 already contained distinct motif subtypes for many TFs. In this release, we took a step further and systematically considered motif subtypes merging ChIP-Seq- and HT-SELEX-derived subtypes, when possible. For many TFs, the motif subtypes were observed only with ChIP-Seq, e.g. for different TF heterodimers binding composite elements, or only with HT-SELEX, e.g. for various combinations of homodimers binding palindromic or tandem repeat sequences. Those cases were kept separately by construction. Of note, we did not try to decouple individual binding sites within composite elements found in ChIP-Seq, and the respective motifs were linked with the particular TFs for which the experimental data were produced.

The CpG methylation-related motif subtypes represented a particularly difficult curation case. On the one hand, in the genomic ChIP-Seq, such motifs usually carry a mix of CpG/TpG possibly arising from several mingled sources: TF-specific binding preference for TGs, CGs, or methylated CGs; varying methylation status of the bound regions; and CpG mutation hotspots. On the other hand, the preference for CpG over TpG and vice versa can be interpreted in HT-SELEX if results from both traditional HT-SELEX and methyl-HT-SELEX (with methylated oligonucleotides) are available. Thus, to keep the HOCOMOCO subtypes comprehensive and consistent, the curators introduced separate CpG and/or TpG motif subtypes when a preference for one or both variants was detected in HT-SELEX/methyl-HT-SELEX experiments. The motifs from HT-SELEX and ChIP-Seq were grouped into a single subtype, when possible, but the information regarding the type of the HT-SELEX experiments contributing to the subtype was kept explicitly.

### Motif quality ratings and subcollections

A popular HOCOMOCO feature is the subjective motif quality or reliability on the A-B-C-D scale where A denotes the most reliable motifs. In the HOCOMOCO v12 core collection, the motif quality ratings were assigned as follows: A for motifs and subtypes found both *in vitro* and *in vivo*, B for those reproducible between individual experiments but *in vitro* or *in vivo* only, and C for all other remaining cases that passed the curation stage. Adapting to the growth of the data and HOCOMOCO usage scenarios, we have excluded from this release suspicious motifs that could have a D quality rating in HOCOMOCO v11. In the v12 core collection, D quality (unclear reproducibility) was assigned to a few motifs belonging to the motif subtypes missed by the v12 pipeline and directly inherited from v11.

In addition to the core collection, we have introduced three subcollections specialized for TFBS prediction *in vivo* (‘v12-invivo’), *in vitro* (‘v12-invitro’), and for rSNP annotation (‘v12-rsnp’), which were based on the benchmarking results from individual data types (see below). In these subcollections, the D rating was assigned to the ‘untested’ motifs of TFs lacking the respective experimental data to perform the specific benchmark.

### Motif benchmarking

To assess the reliability of motif models and select the best-performing model for each motif subtype, we used three sets of computational benchmarks assessing the models' performance in three scenarios: recognizing (I) TFBS bound *in vivo* using ChIP-Seq data, (II) TFBS bound *in vitro* using HT-SELEX data; (III) regulatory SNPs altering TF binding *in vivo* (allele-specific binding in ChIP-Seq (8)) and *in vitro* (SNP-SELEX (43)). Several performance metrics were computed in each category followed by log-rank-sum aggregation across obtained motif ranks for each performance metric, each benchmark, and each test dataset to obtain the final rank of each of the tested motifs for each TF in each of the three benchmarking categories. The top-ranking motifs for each TF formed the three specialized motif collections: HOCOMOCO-invivo (benchmarked on ChIP-Seq), HOCOMOCO-invitro (benchmarked on HT-SELEX), and HOCOMOCO-rSNP. On top of that, another round of log-rank-sum aggregation was performed to obtain the overall best motifs forming the unified HOCOMOCO v12 core collection.

#### ChIP-seq benchmarking

In HOCOMOCO v11 we used independent peak subsets for motif discovery and benchmarking. However, for transcription factors with smaller peak sets this approach reduced the sequence space for motif discovery. In this release, we did not explicitly separate the training data used for motif discovery from the test data for a particular experiment, which introduced some information leakage between model training (motif discovery) and testing (benchmarking). However, (I) for the most studied TFs there were multiple independent experiments to rely on cross-validation across experiments, (II) for many TFs there were both ChIP-Seq and HT-SELEX data available for testing, (III) for a single experiment we used multiple dependent but non-identical peak sets from alternative peak callers, (IV) the dataset of origin was rarely yielding the top-ranked motif in (41) and (V) for poorly studied TFs with a single peak set the performance ratings have limited value in any case.

To reduce the risk of introducing a technical bias in the final collection related to a particular motif performance benchmarking protocol, for HOCOMOCO v12 we used 3 different ChIP-Seq benchmarks.

1. The area under the receiver operating characteristic (auROC) using the Ambrosini *et al.* (41) implementation of the Orenstein-Shamir protocol (44) with the following modifications: up to top 1000 peaks were used as ‘positives’, both downstream and upstream regions were included in the negative set. In addition to auROC, we computed the area under the precision-recall curve (auPRC). For each peak set the benchmark was run twice: the top peaks were selected either by signal value (e.g. peak height) or by statistical significance (e.g. P-value), as reported by the peak calling tools.
2. Asymptotic pseudo-au-logROC as in HOCOMOCO v11 (35) and pseudo-auROC as in HOCOMOCO v10 (45). The benchmark identifies the best PWM hits in 301 bp genomic windows (‘positives’) centered at the peak summits and in random sequences of the same lengths (‘negatives’) following the dinucleotide composition of the positive sequence set. Up to 1000 top peaks from the test data were used, and each benchmark was run twice using the top peaks yielded by score- or significance-based sorting, as above.
3. CentriMo -log-*E*-value of motif centrality (46) measuring how motif hits are located relative to the peak summits. Up to 1000 top peaks from test data were used, and each benchmark was run twice using top peaks yielded by score- or significance-based sorting, as above.

Only peak sets yielding at least one motif that was approved and assigned to one of the motif subtypes during the curation stage were used in benchmarking. In the final motif rankings, for each motif subtype, we used only peak sets (I) comprising ≥100 peaks and (II) for which at least one of the tested motifs reached auROC ≥ 0.6. Subtypes for which no motifs reached auROC ≥ 0.6 were considered not applicable to the ChIP-Seq data and were excluded from the ‘v12-invivo’ subcollection.

Globally, ChIP-Seq benchmark had increased complexity relative to the number of datasets and motifs, thus the most studied TFs with the largest number of ChIP-Seq experiments were bottlenecking the computations. To reduce the computational cost, for the top five of such TFs (CTCF, ESR1, AR, SPI1, FOXA1) we ran the pseudo-auROC benchmark on a randomly selected subset of around 500 datasets. The results were used to pre-rank the motifs and select those ranking from 1 to 250, which were then used in the full-scale benchmarking.

#### HT-SELEX benchmarking

For HT-SELEX, we used the strategy described in (41). The reads from different cycles (for HT-SELEX) were pooled and a maximum of 500 000 randomly sampled unique reads per dataset were used for benchmarking: 10%, 25% or 50% of top-scoring reads were designated as ‘positives’ for each tested PWM. In addition to auROC we also computed auPRC. In the final motif rankings, for each motif subtype, we used only benchmarks where at least one of the tested motifs reached auROC ≥0.6. The subtypes for which no motifs reached auROC ≥0.6 were considered not applicable to the HT-SELEX data and were excluded from the ‘v12-invitro’ subcollection.

#### Benchmarking with regulatory SNPs

With rSNP benchmarking we aimed to identify the motifs suitable for assessing transcription factor binding altered by regulatory single-nucleotide variants. To this end, we employed two data sets: (I) artificial rSNP-carrying oligonucleotides assessed with SNP-SELEX for differential TF binding (43) and (II) sites of allele-specific TF binding *in vivo* from ADASTRA (8). The overview of the benchmarking data is available in Supplementary Table ST5.

**Assessing rSNP prediction with SNP-SELEX data**. We followed the benchmarking protocol described in (47) using the SNP-SELEX data obtained in two batches for 270 and 487 TFs, respectively. Briefly, as in (43), for each TF we distributed TF-bound SNPs between the ‘positive’ set of rSNPs affecting TF binding and ‘negative’ variants. Next, we computed auROC and auPRC of binary classification using the absolute log-ratio of PWM hit *P*-values for the reference and alternative alleles as the predicted classification score. Additionally, for the positives, we used the *P*-value log-ratio to compute Kendal τ_b_ and Pearson ρ against the log-*P*-value reported by SNP-SELEX that reflects the experimentally determined variant-dependent differential binding. In the final evaluation, we included only the test sets with at least ten positive class labels and only the TFs and batch combinations where at least one model reached auROC ≥ 0.6 with both τ_b_> 0 and ρ > 0. The subtypes for which no motifs fulfilled those criteria were considered not applicable to the SNP-SELEX data.

##### Assessing rSNP prediction with ADASTRA allele-specific binding

Compared to SNP-SELEX, the data on allele-specific binding *in vivo* lacks explicit true negatives and does not necessarily reflect direct TF binding, so the true positives, in fact, are mixed with neutral variants. Thus, for each TF, we used HOCOMOCO v11 motif (this annotation was already present in ADASTRA) as a starting filter for selecting candidate SNPs directly overlapping motif occurrences (PWM *P*-value < 0.0005). Of those, we selected the ASB ‘positive’ set (minimal FDR < 0.05 for ChIP-Seq allelic bias towards reference or alternative allele) and the ‘neutral’ set (maximal FDR > 0.5). For the positive set, we computed Kendal τ_b_ and Pearson ρ comparing the log-ratio of PWM *P*-values against the ASB -log-FDR. Additionally, as in ADASTRA (8), we assessed the concordance of the allelic preferences between the predicted (PWM *P*-value log-ratio, log-*P*_Alt_ versus log-*P*_Ref_) and observed (ASB FDR_Alt_ versus ASB FDR_Ref_) difference, and applied the Fisher's exact test to the 2 × 2 contingency table built by counting separately concordant and discordant SNPs in the positive and the neutral ASB subsets. Only TFs with at least 10 ‘positive’ rSNPs and for which at least one model reached Fisher's *P*-value < 0.05 with both τ_b_> 0 and ρ > 0 were considered in the final evaluation. The subtypes for which no motifs fulfilled those criteria were considered not applicable to the ASB *in vivo* data.

In the end, the subtypes inapplicable to both SNP-SELEX and ASB were discarded from the ‘v12-rsnp’ subcollections, and the subtypes inapplicable to any of the available data type were excluded from the core collection.

## Results and discussion

**Figure 2.**
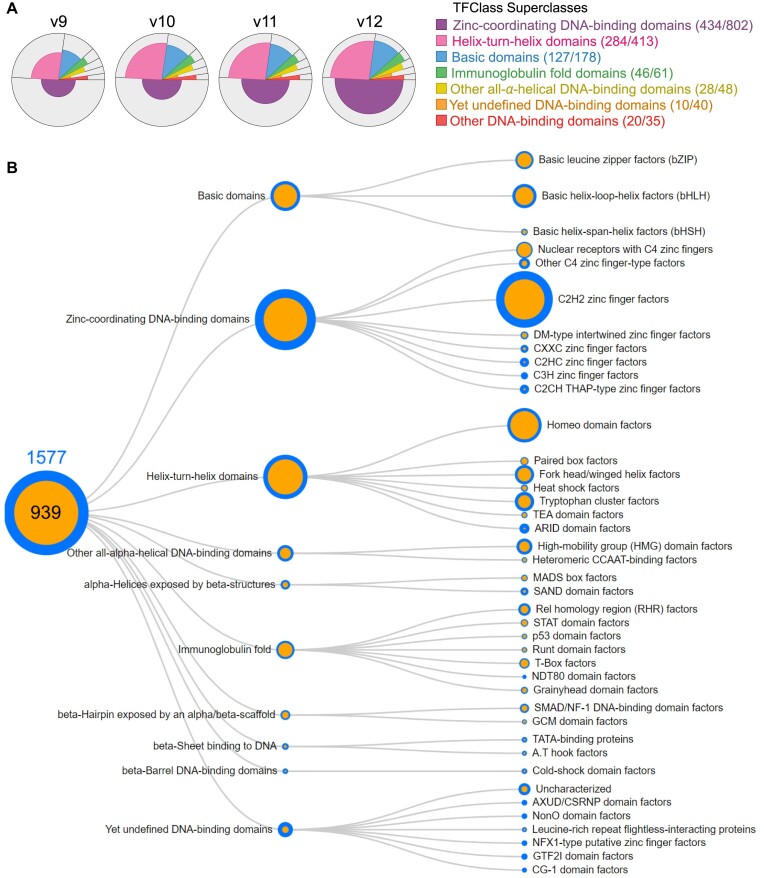
The coverage of different TF classes by HOCOMOCO v12 motifs. **(A)** Improvements in the coverage of transcription factors by HOCOMOCO motifs across the largest DNA-binding domain superclasses, HOCOMOCO from v9 to v12. The pie chart slices denote the contribution of superclasses to the total set of TFs, and the colored parts of the slices denote the fraction of TFs with motifs. The total number of TFs and the number of TFs with HOCOMOCO v12 motifs in each superclass are given in the legend in brackets. **(B)** Relative coverage of different TF classes by HOCOMOCO v12 motifs. Blue: total number of TFs in a superclass or a class, orange: TFs with motifs.

Here we present HOCOMOCO v12, the major update of the curated database of human and mouse transcription factor binding models. HOCOMOCO was constructed by motif discovery in peak sets from 14 183 ChIP-Seq experiments and sequenced reads from 2554 HT-SELEX experiments yielding >400 thousand candidate motifs in total. Of those, around 100 thousand motifs of 949 human TFs were approved, assigned to particular motif subtypes, and supplied to the automated benchmarking pipeline. In result, the HOCOMOCO v12 core collection contains 1443 motifs and covers 949 human TFs, 720 of which have mouse orthologs and 229 are human-exclusive. This is 40% and 60% more TFs than in v11 for the human and mouse, respectively. 241 motifs of 1443 were fully concordant between ChIP-Seq and HT-SELEX for the respective TFs and received the A quality rating, while 819 motifs were concordant between at least two experiments of the same type and received the B quality. Overall, the database update significantly improves the coverage of the motif dictionary across transcription factor families, particularly, with a major increase in binding motifs for TFs with zinc-finger DNA-binding domains (Figure 2A, B; see also the interactive version of the Figure 2B on the HOCOMOCO website). In addition to the core collection, HOCOMOCO v12 provides three extra subcollections fine-tuned for genomic TFBS prediction (v12-invivo), locating sites preferably bound *in vitro* (v12-invitro), and for identifying TFs with altered binding at rSNPs (v12-rsnp).

Due to the increased data volume and improved curation pipeline, in HOCOMOCO v12 we have eradicated most of the unreliable models of D quality which constituted more than a third of the HOCOMOCO v11 full collection. In the core collection, there are now only three D quality motifs inherited from v11 which were approved by curators but not reproduced directly at the motif discovery step of v12. Also, the D quality motifs remain in dedicated subcollections aimed at particular applications of motif libraries (v12-invivo, v12-invitro, v12-rSNP), where motifs untested at the respective target data (HT-SELEX, ChIP-Seq, or rSNPs) are included but explicitly marked by D-s. The v12 core collection includes 380 models of C quality with support from only a single experiment, through expert curation we ensured that these models are consistent either with the DNA binding patterns of the respective TF structural family or known patterns present in other motif collections. Quantitatively, motifs produced by the updated pipeline are better scoring across a diverse set of new benchmarks by design. In the v12 core collection only 11 TFs have motifs inherited from v11, which is probably related to the much larger volume of new data and more rigorous benchmarking setup of the current update. Given the possibly confusing diversity of quality ratings, subtypes, and subcollections, we have designed a flowchart to guide the user in selecting a proper motif collection and a suitable subset of motifs (Supplementary Figure SF1).

With multiple other motif collections covering human and mouse TFs, we consider HOCOMOCO as having its distinctive value for several reasons. First, all motifs were derived by the same motif discovery tool in the same pipeline, making them uniform and comparable across the board. Second, all motifs and multiple motif subtypes were manually curated to reduce redundancy and discard ambiguous patterns and shared artifactual signals. Third, in this version, we provide not only the complete core collection but specifically optimized subsets suitable for different practical needs, from genomic TFBS prediction to designing high-affinity artificial oligonucleotides and regulatory SNP annotation.

Compared to v11, in this release, we do not provide dinucleotide models. Producing and testing these models demanded extra computational expenses for motif discovery due to dramatically increased data volume, and required major changes in the benchmarking protocols, which are not directly applicable to models other than PWMs. Thus, at the current stage, dinucleotide models will remain available in HOCOMOCO v11, while HOCOMOCO v12 can provide a better baseline to derive and test not only dinucleotide PWMs but also more complex models.

The starting ChIP-Seq and HT-SELEX data covered 1022 and 609 human TFs, respectively, but in the end, not all of them are listed in the v12 collection. Unfortunately, our setup does not allow us to state whether these failures have arisen due to the TF itself having limited DNA-binding specificity or technical issues with the original data, data preprocessing, or motif discovery. Data from other experimental technologies may fill this gap in the future.

In this release, we are providing a joint motif collection covering human TFs and their mouse orthologs with a shared set of motifs, motivated by the fact that the TF binding specificities are conserved between human and mouse (30) and for some TFs extend largely even to the level of the fruit fly (48). In HOCOMOCO v11 we cross-validated the motifs between human and mouse and realized that high-performing motifs are performing well across datasets, regardless of the species on which the motif discovery was conducted. Further, in the SNP-SELEX benchmark (47), we observed many cases, where a motif obtained for an ortholog TF of other species outperformed the motif obtained directly from human data. Regarding particular subtypes, there were examples of human- and mouse-exclusive motif subtypes spotted at the curation stage. Yet, in general, the respective TFs were profiled in different cell types and with different antibodies, making it hard to attribute the differences specifically to species and not to technical features of experiments. Thus, in this release, we kept all alternative motifs as species-agnostic motif subtypes, but explicitly annotated subtypes coming exclusively from human or mouse data.

### On redundancy of motif subtypes

**Figure 3.**
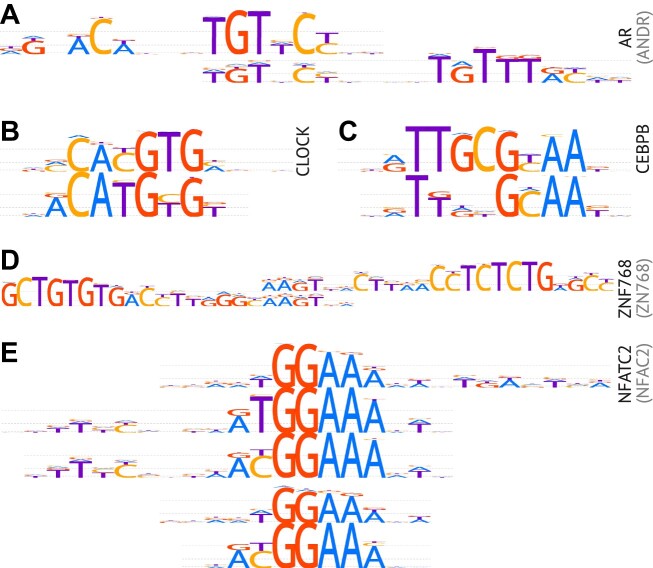
Illustrative examples of motif subtypes included in HOCOMOCO v12. The plots show motif logos, the TF gene symbols are labeled on the right with UniProt entry name prefixes in brackets, if different from the gene symbol. **(A)** The AR palindromic motif (top), and its shorter version making a composite element with FOXA1 (bottom). **(B)** CLOCK motifs derived from ChIP-Seq (top) and methyl-HT-SELEX (bottom). The methyl-HT-SELEX motif prefers TG instead of CG in the central position, which is followed by [C/T]G instead of pure TG in ChIP-Seq. **(C)** CEBPB motifs derived from HT-SELEX (top) and ChIP-Seq (bottom). **(D)** ZNF768 motif subtypes representing overlapping regions of the same repetitive element. **(E)** NFATC2 multiple motif subtypes.

The selection of top-performing motifs from multiple alternatives has been guided by formal criteria of benchmarking measures, which is a major feature of the HOCOMOCO collection. However, the presence of distinct motif subtypes is relying solely on human expert curation. The original idea of including subtypes in HOCOMOCO was to attribute individual motifs to different modes of binding, e.g. distinguish AR palindromic binding sites from AR-FOXA1 (Figure 3A) composite elements. We did not introduce motif subtypes automatically via TF-level motif clustering (49) as many intermediate motifs are blurring the identity of clusters and thus complicating the selection of universal similarity thresholds across the whole range of TFs. Further, this release was designed to be as inclusive as possible and we explicitly included subtypes even with minor motif alterations, if biologically substantiated, e.g. if they arise from different experimental layouts (methyl- vs normal HT-SELEX), different modes of binding (e.g. tandem binding sites in HT-SELEX), or protein-protein complexes (composite elements in ChIP-Seq). Notably, as expected, ChIP-Seq-derived subtypes describing composite elements perform strongly in ChIP-Seq-based benchmarks. Yet, their relative ranks in the core collection vary across TFs, in particular, depending on the composition of the benchmarking data. For example, OCT4-SOX2 composite element is the major first-ranked motif subtype for OCT4 but a secondary motif subtype for SOX2.

A typical example of a relevant but small difference between the subtypes is the CG/TG substitution mostly related to the methylated CpGs. The changeable position arises from two linked but distinct scenarios: (I) TF preferring or avoiding mCpG within the binding sites revealed by methyl-HT-SELEX, and (II) [C > T]G mutation hotspots, leading to depletion of CG pairs in genomic sites and thus affecting ChIP-Seq-derived motifs. The first case is illustrated e.g. by the CLOCK motif subtypes found in HT-SELEX data (Figure 3B). The second case is clearly exhibited for C/EBP motifs (Figure 3C), as C/EBP TFs bind the frequent T-G mismatches at CpGs within its binding sites with increased affinity and by doing so impair base excision repair (11).

A complicated case comes with the repeat-binding zinc-finger proteins, for which ChIP-Seq motif discovery often fails to capture the exact location of the binding site within a longer repetitive element, thus the alternative subtypes do not explain any different modes of binding but rather belong to different parts of the longer consensus, see ZNF768 motifs in Figure 3D.

The diversity of the annotated subtypes in some cases might be excessive, e.g. there are 5 motif subtypes for NFATC2 (Figure 3E), including methylation-specific subtypes and a composite element found in ChIP-Seq. Yet, we nonetheless included all recurrently found motif variants as they could have different practical applications. For example, longer motifs from ChIP-Seq likely capture extended sequence context or cofactor binding patterns and thus are specifically useful for genomic TFBS recognition. Shorter single box motifs might be inefficient in predicting the complete binding sites but useful to decouple TFBS composite elements. Finally, tandem or palindromic motifs from HT-SELEX often represent optimally spaced binding sites with high-affinity and might be suitable to optimize oligonucleotides for binding affinity *in vitro*.

Of note, the motif subtypes in HOCOMOCO v12 are ordered (0, 1, …) according to their performance in benchmarking, thus in the case a user requires a single motif per TF, the first subtype (0) motifs can be safely selected as TF representatives.

### Concluding remarks

Summing up, this release brings HOCOMOCO close to a thousand TFs with reliably described binding specificities. In v12 we have eradicated the legacy non-benchmarked models and models built from low-throughput data, significantly expanded the benchmarking setup, and performed rigorous annotation of motif subtypes. As in the previous release, HOCOMOCO update is accompanied by renewed MoLoTool (motif location toolbox), an interactive JavaScript web application for visualizing motif hits in user-supplied sequences. The online version of our tool for rSNP analysis, PERFECTOS-APE, was also updated to use v12-rsnp collection by default.

We believe HOCOMOCO v12 will serve as a solid knowledge base empowering molecular biology and genetics of gene regulation and also establish the ground for reaching a complete and reliable collection of human and mouse TF motifs in the future.

## Supplementary Material

gkad1077_Supplemental_Files

## Data Availability

The HOCOMOCO v12 database is freely available at https://hocomoco12.autosome.org and https://hocomoco.autosome.org. The HOCOMOCO v12 motif sets and benchmarking results are available at ZENODO [doi:10.5281/zenodo.10012937]. The implementation of ChIP-Seq and HT-SELEX benchmarking protocols is available at GitHub: https://github.com/autosome-ru/motif_benchmarks. The implementation of the HT-SELEX k-mer enrichment estimation is available at GitHub: https://github.com/autosome-ru/kmer-motif-enrichment. The implementation of rSNP-based benchmarking protocols is available at GitHub: https://github.com/autosome-ru/hocomoco_rsnp_benchmarks. The online-only supplementary data are available at the NAR website.
