# Supplementary material for: HOCOMOCO in 2024: a rebuild of the curated collection of binding models for human and mouse transcription factors
Source: Nucleic Acids Res. 2023 Nov 16;52(D1):D154–63. doi: 10.1093/nar/gkad1077 (PMC10767914; doi:10.1093/nar/gkad1077)
Supplement: gkad1077_Supplemental_Files [file gkad1077_supplemental_files.zip › Supplementary Data.docx]

## **HOCOMOCO of 2024: a rebuild of the curated collection of binding models for human and mouse transcription factors**

## Ilya E. Vorontsov, Irina A. Eliseeva, Arsenii Zinkevich, Michael Nikonov, Sergey Abramov, Alexandr Boytsov, Vasily Kamenets, Alexandra Kasianova, Semyon Kolmykov, Ivan S. Yevshin, Alexander Favorov, Yulia Medvedeva, Arttu Jolma, Fedor Kolpakov, Vsevolod J. Makeev, Ivan V. Kulakovskiy

# Supplementary Data

**Supplementary Figure SF1.** A flowchart guide to selecting the appropriate motif subset.

**Supplementary Table ST1.** HOCOMOCO master list of transcription factors.

**Supplementary Table ST2.** List of ChIP-Seq experiments from GTRD used in the study.

**Supplementary Table ST3.** List of HT-SELEX experiments used in the study.

**Supplementary Table ST4.** Motif discovery command line parameters.

**Supplementary Table ST5.** Overview of rSNP benchmarking data.
