# Supplementary figures and images for: HOCOMOCO in 2024: a rebuild of the curated collection of binding models for human and mouse transcription factors
Source: Nucleic Acids Res. 2023 Nov 16;52(D1):D154–63. doi: 10.1093/nar/gkad1077 (PMC10767914; doi:10.1093/nar/gkad1077)

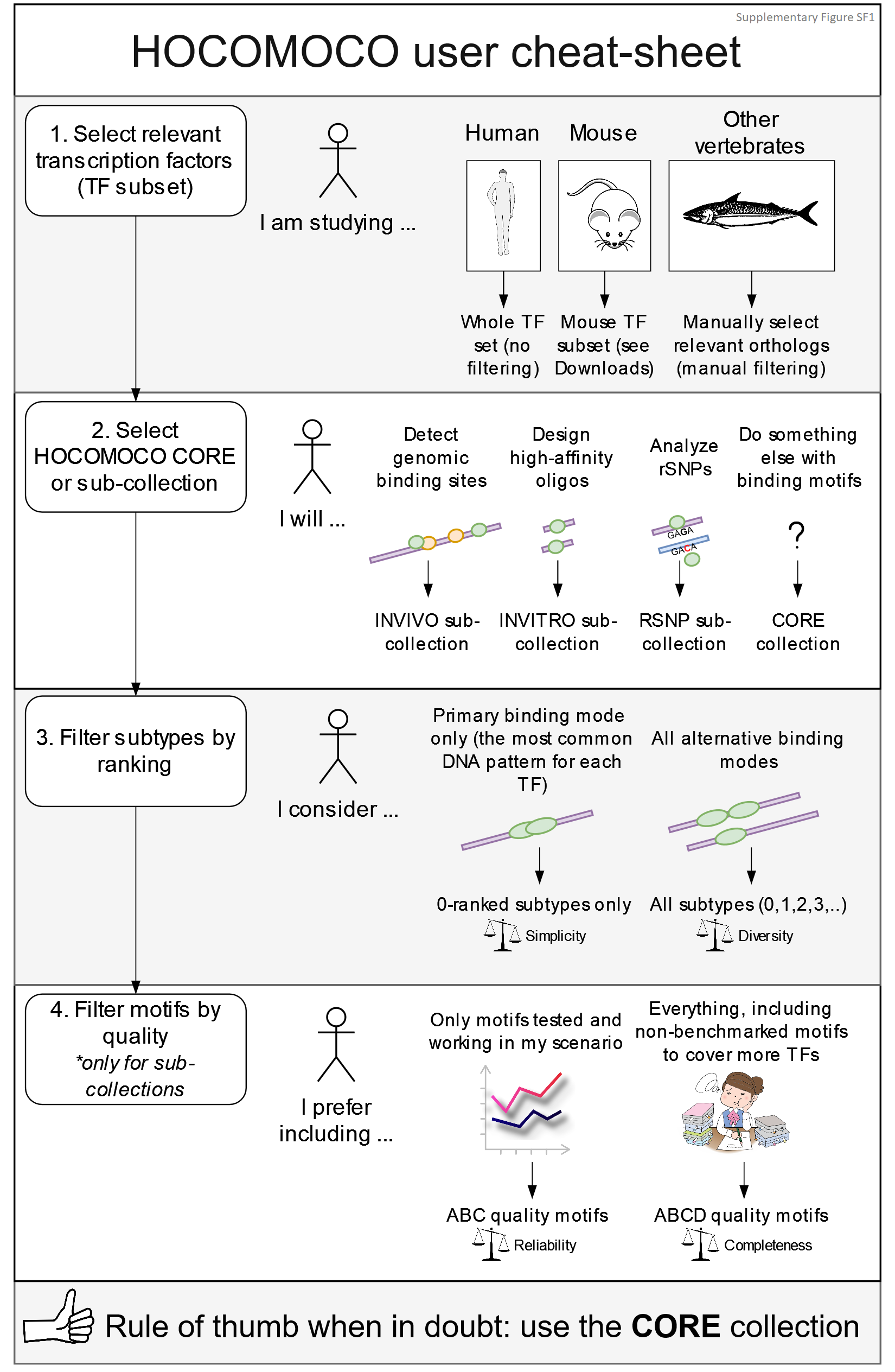

Supplement: gkad1077_Supplemental_Files [file gkad1077_supplemental_files.zip › Supplementary Figure SF1 - wcaption.png]
